# Executive functioning in TBI from rehabilitation to social reintegration: COMPASS ^goal,^ a randomized controlled trial (grant: 1I01RX000637-01A3 by the VA ORD RR&D, 2013–2016)

**DOI:** 10.1186/s40779-015-0061-2

**Published:** 2015-11-30

**Authors:** Alexander V. Libin, Joel Scholten, Manon Maitland Schladen, Ellen Danford, Nawar Shara, Walter Penk, Jordan Grafman, Linda Resnik, Dwan Bruner, Samantha Cichon, Miriam Philmon, Brenda Tsai, Marc Blackman, Alexander Dromerick

**Affiliations:** Mental Health Service, Washington DC VA Medical Center, 50 Irving Street, NW, Washington, DC 20422 USA; MedStar National Rehabilitation Hospital, 102 Irving Street, NW, Washington, DC 20010 USA; Physical Medicine and Rehabilitation Service, Washington DC VA Medical Center, 50 Irving Street, NW, Washington, DC 20422 USA; Research Service, Washington DC VA Medical Center, 50 Irving Street, NW, Washington, 20422 DC USA; MedStar Health Research Institute, 6525 Belcrest Rd #700, Hyattsville, MD 20782 USA; Texas A&M College of Medicine, 8447 TX-47, Bryan, TX 77807 USA; Rehabilitation Institute of Chicago, 345 E Superior St., Chicago, IL 60611 USA; Research Service, Providence VA Medical Center, 830 Chalkstone Ave, Providence, RI 02908 USA

**Keywords:** Executive function, Traumatic brain injury, Goal-setting, Community re-integration, Veterans, Randomized controlled trial, Manualized psychosocial intervention

## Abstract

**Background:**

Traumatic brain injury is a major health problem that frequently leads to deficits in executive function. Self-regulation processes, such as goal-setting, may become disordered after traumatic brain injury, particularly when the frontal regions of the brain and their connections are involved. Such impairments reduce injured veterans’ ability to return to work or school and to regain satisfactory personal lives. Understanding the neurologically disabling effects of brain injury on executive function is necessary for both the accurate diagnosis of impairment and the individual tailoring of rehabilitation processes to help returning service members recover independent function.

**Methods/design:**

The COMPASS^goal^ (Community Participation through Self-Efficacy Skills Development) program develops and tests a novel patient-centered intervention framework for community re-integration psychosocial research in veterans with mild traumatic brain injury. COMPASS^goal^ integrates the principles and best practices of goal self-management. Goal setting is a core skill in self-management training by which persons with chronic health conditions learn to improve their status and decrease symptom effects. Over a three-year period, COMPASS^goal^ will recruit 110 participants with residual executive dysfunction three months or more post-injury. Inclusion criteria combine both clinical diagnosis and standardized scores that are >1 SD from the normative score on the Frontal Systems Rating Scale. Participants are randomized into two groups: goal-management (intervention) and supported discharge (control). The intervention is administered in eight consecutive, weekly sessions. Assessments occur at enrollment, post-intervention/supported discharge, and three months post-treatment follow-up.

**Discussion:**

Goal management is part of the “natural language” of rehabilitation. However, collaborative goal-setting between clinicians/case managers and clients can be hindered by the cognitive deficits that follow brain injury. Re-training returning veterans with brain injury in goal management, with appropriate help and support, would essentially treat deficits in executive function. A structured approach to goal self-management may foster greater independence and self-efficacy, help veterans gain insight into goals that are realistic for them at a given time, and help clinicians and veterans to work more effectively as true collaborators.

## Trial registry

*Registry:* ClinicalTrials.gov Identifier - NCT01816061.

*Dataset:* See Table 1 for items from the World Health Organization Trial Registration Data Set.

**Table 1:** Trial registration data set

Data category	Information
Primary registry and trial identifying number	ClinicalTrials.gov NCT01816061
Date of registration in primary registry	March 19, 2013
Secondary identifying numbers	I01RX000637-01A3 D0637-R
Source(s) of monetary or material support	United States Department of Veterans Affairs Rehabilitation Research and Development Service
Primary sponsor	United States Department of Veterans Affairs Rehabilitation Research and Development Service
Contact for public queries	AVL [Alexander.Libin2@va.gov], ED [Ellen.Danford@va.gov]
Contact for scientific queries	AVL [Alexander.Libin2@va.gov]
Public title	Executive Functioning in TBI from Rehabilitation to Social Reintegration: COMPASS
Scientific title	Executive Functioning in TBI from Rehabilitation to Social Reintegration: COMPASS
Countries of recruitment	United States of America
Health condition(s) or problem(s) studied	TBI
Intervention(s)	Experimental—Fifty-five participants in the intervention group will receive eight goal self-management sessions. Control—Increased hours of patient-provider interactions.
Key inclusion and exclusion criteria	Ages eligible for study: 18–55 Sexes eligible for study: Both Accepts healthy volunteers: Yes Inclusion and Exclusion Criteria: See Table 2.
Study type	Interventional Allocation: Randomized Intervention Model: Parallel assignment Masking: Single blind (outcome accessor) Primary Purpose: Treatment Phase 0
Date of first enrollment	December 2014
Target sample size	110
Recruitment status	Recruiting
Primary outcome(s)	Change in Baseline in CRIS assessment at 2 months and 5 months. Change in Baseline in FrSBe assessment at 2 months and 5 months.
Key secondary outcomes	N/A

## Protocol version

Issue Date: 21 Aug 2015

Protocol Amendment Number: 06

Author(s): AVL, JS, MMS, ED, NS, WP, JG, LR, DB, SC, MP, BT, MB, AD.

Revision Chronology (Institutional Review Board (IRB))

*27 Jan 2014* Original.

*08 Aug 2014* Amendment 01: Primary reason for amendment – Submission of COMPASS^goal^ manual; updated recruitment procedures; addition of study staff.

*24 Sep 2014* Amendment 02: Primary reason for amendment—Updated assessments to reflect DSM-V [1].

*11 Dec 2014* Amendment 03: Primary reason for amendment—Updated assessments based on access issues; clarified control group protocol; changes to consent forms; addition of study staff.

*26 Jan 2015* Amendment 04: Primary reason for amendment—Updated manual and consent forms, addition of study staff.

*01 Apr 2015* Amendment 05: Primary reason for amendment—Updated recruitment flyers; addition of retention letters.

*21 Aug 2015* Amendment 06: Primary reason for amendment—Removal of assessment; updated consent forms; addition of recruitment brochure.

## Roles and responsibilities

### Contributorship

AVL conceived of the study. JS, MMS, NS, WP, MP, BT, MB, and AD participated in the initial study design, and AVL, JS, MMS, ED, DB, and SC led the implementation of the study. NS guided statistical implementation and is conducting primary statistical analysis. All authors contributed to the refinement of the study protocol and edited and approved the final manuscript.

### Sponsor contact information

Trial Sponsor: United States Department of Veterans Affairs Rehabilitation Research and Development Service.

Contact Name: Patricia Dorn, PhD

Address: 810 Vermont Avenue, NW (10P9R)

Washington, DC 20420

Telephone: 202-443-5756

Fax: 202-495-6197

### Sponsor and funder

This funding source reviewed the study design prior to implementation but will have limited to no role during its implementation, data analysis, and results publication.

### Committees

No committees are involved in the execution of this study.

## Background

### Background and rationale

**Fig. 1:**
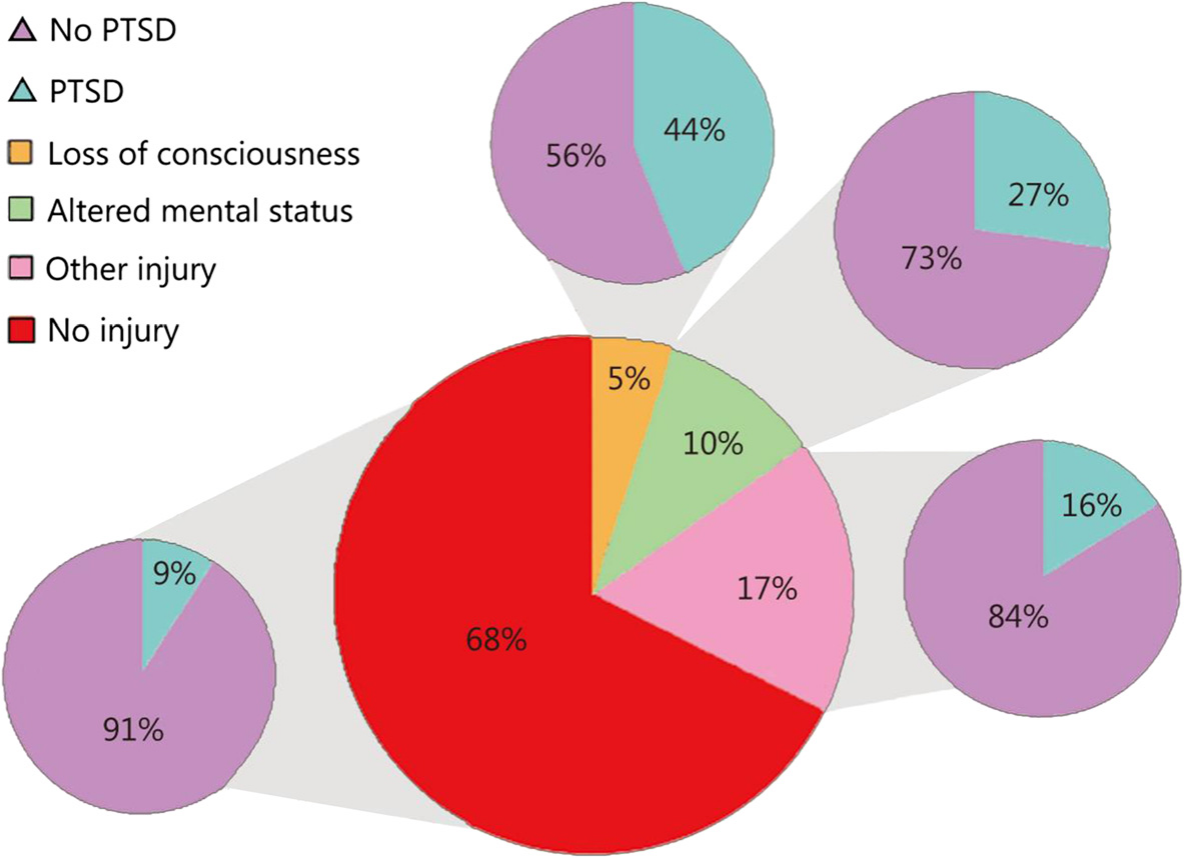
Data extracted from article titled Mild traumatic brain injury in U.S. Soldiers returning from Iraq [105] showing PTSD prevalence in 2,517 Iraq conflict veterans by reported injury

Traumatic brain injury (TBI) is a direct cause of long-term cognitive disability in returning United States (U.S.) veterans [2]. TBI is also an established risk factor for psychological health and community re-integration [3, 4]. Studies emphasize the dramatic effects of neurological injuries among active duty troops serving in conflicts in Afghanistan and Iraq. (see Fig. 1).

There is mounting evidence that suggests that executive dysfunction due to central nervous system (CNS) insult causes both short-term and long-term consequences, resulting in poor goal-directed behavior [3, 5–8] and significant decreases in independent functioning in both soldiers and returning veterans. Given the necessity for operational effectiveness in the battlefield environment, executive function symptoms become significant for the military because they jeopardize active duty troops’ capacity for crucial decision making [9, 10]. At the same time, while remaining undetected, neurological problems cause poor self-management skills, resulting in maladjustment and a low quality of life in returning military veterans [3, 11, 12]. In this study, we employ the definition of TBI by the U.S. Individuals with Disabilities Education Improvement Act of 2004, i.e., “…an acquired injury to the brain caused by an external physical force, resulting in total or partial functional disability or psychosocial impairment, or both, that adversely affects a person’s professional and educational performance in one or more areas, such as cognition; language; memory; attention; reasoning; abstract thinking; judgment; problem-solving; sensory, perceptual, and motor abilities; physical functions; information processing; and speech. The term does not apply to brain injuries that are congenital or degenerative, or to brain injuries induced by birth trauma [13].” The exposure to stressors from combat, including explosive blasts and loss or injury to the self or to comrades, can lead to significant problems when transitioning back to civilian life post-combat [14, 15]. Due to advances in medicine and body armor, soldiers are surviving blasts or explosions that may have previously resulted in severe injury or death during combat. Following the U.S. defense causality report in November 2009, the Institute of Medicine updated its statistics to show that “fatality-to-wounded ratios have been 1:5.0 for… [conflict in Afghanistan] and 1:7.2 for…[conflict in Iraq] [14, 16] compared with 1:2.6 in Vietnam and 1:1.7 in World War II [14, 17].”

Many veterans returning from the wars in Afghanistan and Iraq may have experienced TBI [12], the significance of which is underscored by a national study undertaken by the RAND (Research ANd Development) Corporation commissioned by the U.S. Office of the Secretary of Defense for Health Affairs to gauge the effect of TBI on the lives of veterans and their families [18]. Even if there are no other co-existing physical impairments, TBI and post-traumatic stress disorder (PTSD) are enough to significantly hinder a veteran’s successful progression into active community participation and employment. Physical factors affecting community re-integration in veterans with polytrauma and TBI include pain, PTSD-related anger, and depression. Among the psychosocial factors that affect community functioning in returning veterans are social isolation, poor problem-solving of everyday difficulties, and a lack of motivation to change.

#### Executive functioning system in TBI: a multifactorial model

Executive dysfunction is the core condition underlying neurologic impairments resulting from CNS insult, such as TBI [2], dual TBI-spinal cord injury [19], and stroke [20], and it is a distinct feature of CNS degenerative disorders such as Parkinson’s disease [21]. As a central clinical syndrome, executive functioning is defined as a network of processes that are responsible for initiating, guiding, and regulating psychomotor, cognitive, emotional, and behavioral functions, particularly during active and novel problem-solving [7, 22, 23].

**Fig. 2:**
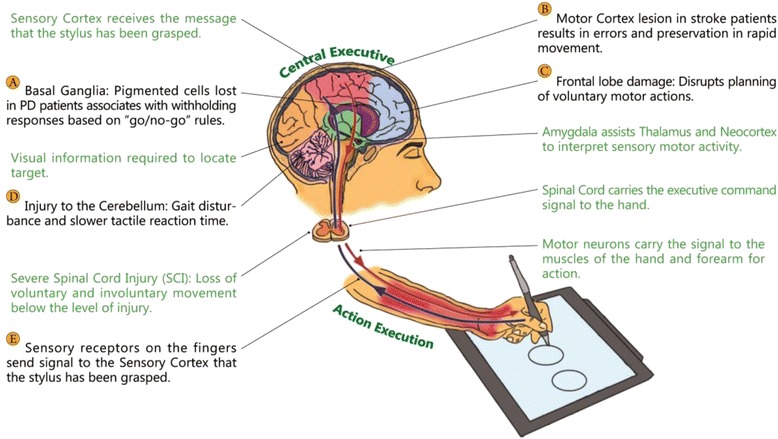
Central-peripheral dysregulation in Executive Functional System(EFS)

Executive processes are thought of as part of the system that acts in a supervisory capacity in the overall brain hierarchy [24] and provides for purposeful, goal-directed behavior [25–27]. A plethora of neurologic and behavioral data demonstrates that individual performance processes are deeply involved with changes in what can be called the executive functional system (EFS) (see Fig. 2*)* [28, 29].

Recent studies emphasize that executive processes do not have a single neuroanatomical representation in the CNS [6, 30]; rather, they present as patterns that merge different brain structures (e.g., the brain’s prefrontal zone and limbic system) and their peripheral counterparts (e.g., motor apparatus). Existing interlinkages in the brain-peripheral system are currently explored by measuring various aspects of executive functioning through standardized neuropsychological assessments, magnetic-resonance imaging and a more precise assessment, diffusion tensor imaging [31]. The conceptual definition of the EFS proposes two major subsystems: the *central executive subsystem* [32], which includes higher-level processes, such as selective attention, working memory, and decision-making capacity [6], and the *peripheral executive subsystem*, composed primarily of psychomotor processes associated with central executive performance [33, 34]. Executive functioning can break down at any stage in the behavioral sequence, be it volition control, planning, purposeful behavior, or effective performance [35]. Deficiencies in self-initiated behaviors may result from neurological damage to the frontal-subcortical or fronto-limbic circuitry (see areas C and D, Fig. 2) [36, 37], to the right hemisphere, or in diffuse neurologic conditions (see area A, Fig. 2) [38]. The dorsal prefrontal cortex is critical to allocating attentional resources involved with working memory tasks [39] or to the attentional controller [40] – a system responsible for maintaining and switching attention [41].

Motor performance is an instrumental component of the EFS [33, 34]. In the classic methodology of motor examination tasks developed by Luria and his successors [41, 42], the ability to copy hand movements paced by a metronome [43] was found to be sensitive to frontal damage and to temporal lesions (see area C, Fig. 2). The inability to move rapidly through a repetitive or mixed-movement sequence, combined with errors and perseverations, was found to be characteristic of patients with left hemisphere lesions (see area B, Fig. 2) [44]. Motor regulation deficits are often associated with withholding responses [45], based on a “go/no-go” rule, in which a subject should respond to only one of two presented signals (see area A, Fig. 2) [26].

Patients with subcortical involvement display executive dysfunction that includes impairments in cognitive flexibility, memory recall, and psychomotor slowing [46–48]. Impairment of executive processes often presents a major challenge in an individual’s ability to perform activities of daily living [49], to manage their disability [50], and to reintegrate into the community [51].

#### Executive System: TBI, PTSD and community re-integration

There is an overlap of symptoms between TBI and PTSD. This issue is most pertinent in the chronic TBI population, where there are higher rates of PTSD. Sustaining any type of physical injury is known to increase a person’s risk for PTSD [52]. There are several symptoms that are found in *both* PTSD and TBI, such as deficits in attention and memory, irritability, and sleep disturbance. However, in the acute assessment of TBI, distinguishing symptoms such as headache, dizziness, balance problems, and nausea/vomiting may help distinguish TBI from PTSD. Another distinguishing factor is the history that is obtained from patients about the course of events before, during, and after the traumatic event. Loss of consciousness and post-traumatic amnesia are less common in PTSD and is the distinguishing historical factor to diagnose mild and moderate TBI.

Research has suggested a range of post-injury cognitive, somatic, and behavioral symptoms, including headache, anxiety, dizziness, and memory difficulties immediately following TBI [47, 53, 54] and decreased educational attainment and limitations in work performance [55, 56]. The effects of cognitive and behavioral impairment on independence and societal participation following TBI are well established. At the same time, the gaps between research and everyday life continue to exist. A U.S. Institute of Medicine report on the needs of veterans returning from Afghanistan and Iraq indicates, “little research has been conducted to evaluate whether service members who undergo third location decompression (that is, for service members to have time with their comrades and peers in a restful situation and prepare themselves for going back to their families and communities) have better outcomes than those who do not [14].”

Strengthening the community integration of veterans with TBI requires a collaborative effort bringing together veterans and their families, Veterans Health Administration (VHA) case managers and social workers, and the broader research community to address barriers that prevent veterans with serious injures such as TBI from effectively pursuing life opportunities that are available to others. In one of the first national surveys of veterans in Afghanistan and Iraq enrolled in the Department of Veterans Affairs (VA) health care system who saw combat, Sayer and colleagues (2010) [57] explored the prevalence and types of community re-integration problems veterans faced and assessed preferences for interventions to promote adaptation to civilian life. Stratified Poisson regression was used to determine whether the number of community reintegration problems and the number of services of interest were associated with the presence of probable PTSD, gender, or race. An estimated 25 % to 56 % of the population had some difficulty to extreme difficulty in the social functioning, productivity, community involvement, and self-care domains. At least one third reported divorce, dangerous driving, increased substance use, and anger control problems since returning from deployment. An estimated 41 % had probable PTSD, and each type of reintegration problem was more prevalent among veterans with probable PTSD. The vast majority (96 %) of Afghanistan/Iraq veterans expressed interest in services to help them readjust to civilian life. The most commonly preferred ways to receive reintegration service or information was at a VA facility, through the mail, and over the internet. Interest in self-help techniques and yoga/meditation was particularly common [58]. Penk and colleagues (2010) [59], in their VHA comparative effectiveness study, discussed the need for research that identifies ways to aid veterans with dual diagnoses attain competitive jobs. Community re-integration tools were found to be key to the effectiveness of employment programs providing veterans not only with income-earning work but also with skills to help them secure employment.

#### International classification of function, disability, and health (ICF)

In the Community Participation through Self-efficacy Skills Development: COMPASS^goal^ study, we consider function and disability, as well as activity and participation (including employment), based upon the widely used ICF framework [60] developed under the auspices of the World Health Organization. Through this inclusive approach, the study team documents the natural process of recovery in TBI, including the occurrence of accelerated recovery, thereby addressing the needs of people with chronic TBI and providing potential benefits. At each assessment time point, participants (and their families and/or caregivers, if appropriate) will complete validated, standardized scales that capture outcomes in four central domains, as defined by the ICF model: function, health, participation, and employment. The ICF presents an interaction of several basic concepts in disability that is widely used as a methodological tool for studying physical disability in general and TBI in particular. Relevant to the present project’s conceptual integration is the ICF proposed model of contextual (personal and environmental) factors as they relate to the individual health condition [60].

#### COMPASS^goal^ conceptual framework

COMPASS^goal^ aims to address the unmet needs of veterans of conflicts in Afghanistan and Iraq for successful return to civilian life [9, 11, 14, 15]. Employing the proven elements from self-management training and other goal-management programs successfully implemented with civilian clinical populations, COMPASS^goal^ guides participating veterans, their families, and interventionists through the process of negotiating goals, establishing hierarchies of long-, mid-, and short-range goals, and developing individualized, measurable ways of tracking progress. A skill that is emphasized is the ability to break distal, or long-term, goals into proximal or short-term goals and to use one’s performance on proximal goals to modify the distal ones as necessary [61]. Other specific components incorporated into the COMPASS^goal^ protocol include three over-arching self-regulation strategies that have been shown in meta-analyses to be effective: goal manipulations, arousal management (both relaxation and increasing arousal/motivation), and cognitive self-regulation, which includes self-monitoring, evaluating, and adjusting performance to meet a selected standard [62]. A meta-cognitive technique called “mental contrasting”—simultaneously considering both the positive aspects of the goal state and the negative aspects of one’s current state— is also employed [63]. Finally, self-monitoring skills are incorporated because these also have a strong evidence base [62].

#### Choice of comparators

Interdisciplinary care is essential to TBI rehabilitation. Per VA guidelines, many veterans receiving care for TBI-related problems are also followed by a case manager and an interdisciplinary team [11, 64]. As part of this care, veterans frequently answer the Mayo-Portland Adaptability Inventory—4 Participation Index (M2PI) [65]. The test is a self-report by the person with TBI. The M2PI is currently used by the Washington DC VA Medical Center (DC VAMC) TBI clinic team when the veteran is initially evaluated in a team meeting, when the veteran is discharged from team treatment, and three to six months post-discharge. Thus, the administration of the M2PI mimics interactions with a case manager. The M2PI functions as a checklist for the research team and fulfills the role of increased interactions with a case manager. It encourages veterans to reflect upon their community integration and offsets the possible effects of the additional time and attention devoted to participants in the COMPASS^goal^ group models.

### Objectives

The main objective of the COMPASS^goal study^ is to determine whether veterans who have executive dysfunction due to mild traumatic brain injury (mTBI) will benefit from a novel goal self-management intervention, COMPASS^goal^, compared to veterans who receive case management support that represents the current standard of care enhanced by an increased number of communications with VA staff.

The specific aim and hypotheses of the COMPASS^goal^ study are:

- Study Specific Aim 1: To develop, implement, and evaluate a new goal self-management intervention (COMPASS^goal^) for veterans with executive dysfunction due to mTBI and to investigate how executive functioning is linked to the performance of everyday tasks and community functioning.
- Study Hypothesis 1: Participants in the COMPASS^goal^ group will have higher community integration scores over time than participants in the supported discharge group matched on executive dysfunction score.
- Study Hypothesis 2: Individuals’ psychosocial profiles (emotional status, resilience, and level of PTSD) will mediate the responsiveness to the COMPASS^goal^ intervention, measured through standardized experimental performance of everyday tasks, in veterans with impaired executive function due to mTBI.

### Trial design

The COMPASS^goal^ study is designed as a randomized, controlled, single blind (outcome accessor) efficacy study with two parallel groups. The primary endpoint of the study occurs with final data collection at time point three, three months after the completion of either group. Participants will be randomly assigned to either control or experimental groups with a 1:1 ratio based on Wei’s Urn randomization algorithm [66]. Over the three-year course of the COMPASS^goal^ project, we will screen, consent, and baseline 110 veterans, aged 18–55 years, who have been diagnosed with mTBI. All participating veterans undergo a battery of tests measuring executive function, real-world performance, TBI self-efficacy, emotional status and PTSD, community integration, and quality of life. Each potential participant receives additional screening of TBI and executive dysfunction to determine intervention eligibility. Each participant also receives a neuropsychological interview, and COMPASS^goal^ investigators discuss each participating veteran with his/her VA case manager, as applicable. Subsequently, veterans are randomized to intervention and control groups. The former receives the COMPASS^goal^ self-management intervention developed to support vulnerable transitions identified during the first 6 months of the project. The latter receives focused, but standard-of-care, support from the VA TBI Research team. Intervention-group veterans receive weekly eight one-on-one goal management sessions of up to 90 minutes over a period of two months. Veterans in both the intervention and control groups receive assessments before, directly following, and three months following the completion of the COMPASS^goal^ intervention or supported discharge process. The data will be modeled longitudinally and on multiple levels to identify vulnerable transitions and predictors of community integration/participation outcomes. The findings will form the basis for clinical practice guidelines.

The three-year, multi-phase study explores two inter-related hypotheses. Hypothesis 1 is explored through a randomized controlled trial (RCT) that tests the efficacy of a newly developed intervention, COMPASS^goal^, in 110 young to middle-aged veterans with mTBI assigned to intervention goal self-management or supported discharge groups. Hypothesis 2 is aimed at studying the multilevel relationships between four sets of variables (neurological, psychological, behavioral, and social) measured repeatedly for the duration of the project.

## Methods

### Participants, interventions, and outcomes

#### Study setting

**Fig. 3:**
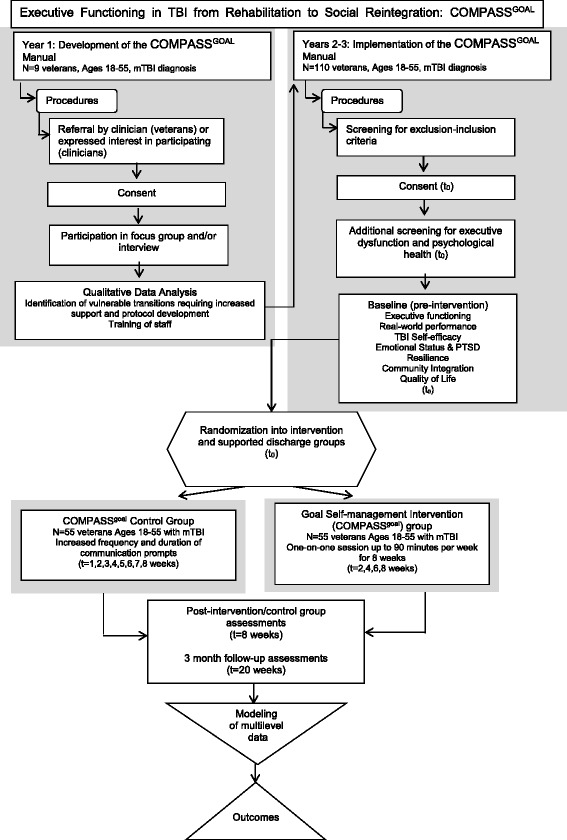
COMPASS^goal^ Study Algorithm

The study procedures take place at the DC VAMC TBI/polytrauma clinic either face-to-face or by phone (see Study Algorithm, Fig. 3). This urban clinic evaluates 150–200 new veterans per year who are identified with a positive TBI screen. Currently, the positive TBI diagnosis rate is approximately 50 % for the DC VAMC site. In addition, other veterans with known TBI are referred to the clinic, resulting in an additional 50–100 referrals per year. The frequency of follow-up visits is determined on an individualized basis. In addition to the veterans with TBI who are seen at the DC VAMC each year, many veterans hospitalized at the Richmond (Virginia) Polytrauma Rehabilitation Center and discharged to the adjacent Washington DC Metropolitan Area are subsequently seen in the DC VAMC TBI clinic. All veterans who consent to the study are examined using the Frontal Systems Behavior Scale (FrSBe) to measure executive function. VA expert clinician informants estimated that 50–75 % of veterans with mTBI have comorbid impaired decision-making/executive dysfunction and will be eligible and willing to participate in the study (*personal communication with Kelly McCarron, PsyD and Sheree Gordon, MSN*).

We make every effort to balance the sample by age, gender, and education following the study inclusion–exclusion criteria. Based on previous studies conducted with the VA population in the Greater Washington DC Metropolitan Area, we expect that approximately 10 % of the study participants will be female, and 70 % will be African-American. The total number of study participants (N = 110) accounts for a 20 % attrition rate in a neurologically disabled population due to their health condition and related problems.

#### Eligibility criteria

##### Participants

One hundred ten medically stable individuals with residual deficits due to mTBI are recruited from the TBI clinics at the DC VAMC over the three-year period of the study and are enrolled into the study according to the inclusion and exclusion criteria set forth in Table 2.

**Table 2:** COMPASS^goal^ Inclusion and exclusion criteria

	Inclusion criteria	Exclusion criteria
COMPASS^GOAL^: community re-integration study	TBI of at least mild severity using criteria related to disturbance of consciousness (VHA TBI Comprehensive Evaluation screening tool) [67]	Inability to provide informed consent and no proxy available
	Obtained informed consent	Severe impairment of language or day-to-day memory that would preclude participation in a verbal-based therapy
	Males or females of working age, between the ages of 18 and 55	Life expectancy of less than 36 months
	Medically stable with physician approval to participate	Severe multiple trauma (judged by the attending physicians and/or investigators as too severe for participation in this study), such as severe burns, serious organ damage, amputations, or multiple fractures
	Ability to comprehend and communicate in English at a 6th-grade level	History of substance abuse severe enough to cause neurologic damage, pre-morbid history of neurologic disease (e.g., stroke)
	*Executive dysfunction as identified by the FrSBe* [68] *and/or other study assessments (see Table* 3 *)*	Prior history of known bipolar disorder or schizophrenia or severe psychiatric illness as confirmed by medical records and/or clinical judgment or M.I.N.I. assessment [69–72] if no clinical judgment is on record

The severity of TBI will be determined by the DC VAMC attending clinician using the VHA TBI Comprehensive Evaluation electronic template, questions 6–8 [67]. Prior diagnosis by the attending physician through the electronic template will be confirmed through a review of medical records or an interview with the attending physician upon enrollment in the study. The FrSBe is used to identify individuals with differing levels of executive dysfunction. The operational definition of executive dysfunction/inclusion into the study is based on both clinical diagnosis by a study physician and a standardized executive dysfunction measure, the FrSBe score, such that a total score or any of the 3 subscale scores >1 SD compared to the normative score would indicate executive dysfunction sufficient to include in the study [68]. Prior history of known bipolar disorder, schizophrenia or severe psychiatric illness are determined first by a review of the medical history, and in the absence of clinical psychiatric assessment, the M.I.N.I. International Neuropsychiatric Interview (M.I.N.I.) assessment is completed by study staff. The M.I.N.I. has demonstrated reliability and validity compared to structured clinical interviews [69–72]. We believe both methods will adequately screen potential participants for possible severe mental health confounders.

##### Interventionists

For purposes of this study, the interventionist will be a Master’s level case worker who completed training regarding the military population with practicing psychologists, case managers, and physicians. We selected the interventionist to ensure that the COMPASS^goal^ manual could be utilized by clinicians of diverse training levels, following initial testing.

The research staff underwent intensive training during months 9–11 of Year 1 and received ongoing relevant education. The principal investigators assume the responsibility of supervision of the COMPASS^goal^ staff involved with intervention delivery and also provide mentoring regarding specific medical, family, and community issues as needed. Initially, daily meetings among the research team will be held to address operational, procedural, and scheduling issues. Staff meetings take place weekly to address difficult cases and to share successful management strategies and new resources.

To successfully implement the proposed intervention as a complementary service to participating veterans with TBI, the study staff employs the framework developed in the VHA Handbook [11] 1172.04, Physical Medicine and Rehabilitation: Individualized Rehabilitation and Community Re-Integration Care Plan (2010). This VHA Handbook defines procedures for development and implementation of the Individualized Rehabilitation and Community Reintegration Care Plan for veterans and Military Service members who receive inpatient or outpatient rehabilitative care for functional deficits or needs related to TBI and polytrauma. The COMPASS^goal^ protocol interplays at each phase of development and implementation with the procedures for Individualized Rehabilitation and Community Re- Integration Care Plans specified by the VHA Handbook and carried out at the DC VAMC.

#### Intervention

##### Manual development

Aim 1 includes the development and implementation of an innovative treatment program, COMPASS^goal^, to mentor veterans with executive dysfunction due to mTBI in evidence-based techniques for implementing goal self-management. This process is guided by a systematic, written protocol, a COMPASS^goal^ manual for interventionists, and patient-friendly materials customizable to support individual treatment of veterans with mTBI who participate in the study. The COMPASS^goal^ manual guides interventionists in the process of working with veterans with mTBI to define and track progress towards goals throughout the study timeframe.

Prior to the finalization of the COMPASS^goal^ manual, focus groups and semi-structured interviews were held with veterans with mTBI from a variety of backgrounds and with VA clinicians from practice areas such as case work, occupational, physical, speech, and recreation therapy and psychology and counseling, representative of how the VA delivers services nationwide. The purpose of these interviews and focus groups was to assure that the content and format of the COMPASS^goal^ intervention was broadly relevant to both veterans’ goals in the context of their lived experiences and to the guidelines of clinicians’ practice across VA service environments. Interviews and focus groups were completed from March-August 2014.

##### Intervention and supported discharge group

Veterans are randomized to the intervention or control groups. The former receives the COMPASS^goal^ self-management intervention developed to support vulnerable transitions identified during the recruitment assessment and interviews/focus groups. Each client in the intervention group receives up to 90 minutes per week of treatment either face-to-face or over the phone, which follows the written protocol (COMPASS^goal^ Manual). This treatment is conducted by trained research staff. Studies have shown the effectiveness of both telephone and face-to-face administration of psychosocial and rehabilitative interventions [73–76]. Because the COMPASS^goal^ intervention is experimental, there are no data to show that the two approaches are comparable for this specific intervention. However, care is often delivered at a distance in the VA system through various modes of telemedicine, due in part to distance barriers [77]. Hence, diverse care delivery practices are increasingly normative in the study population. We anticipate comparable effectiveness for those receiving the intervention remotely and face-to-face, but we acknowledge that differences may arise, and the modalities will be analyzed as a separate variable.

In the course of weekly sessions, each client develops his or her goal planner. The planner is conceived as a portfolio that contains the written materials generated during goal planning with various tabs for long- mid-, and short-term goals and activities related to them, and all materials are designed collaboratively with each client for goal tracking. Depending on the individual client’s comfort with and access to technology, the materials are managed online through a secure portal. The goal planner is updated weekly during intervention sessions.

Goal sessions with coaches are audiorecorded to better inform the researchers about the effectiveness of the interventions. Significant others (spouses or other close friends/relatives) are invited to participate in the assessments and the sessions at the discretion of each participating veteran to provide support outside of treatment for implementing the procedures learned in each session.

Participants randomized into the supported discharge group serve as controls for the intervention group and do not receive the COMPASS^goal^ intervention. Each control group participant receives additional prompts to address just-in-time concerns, as documented in their treatment plans. This strategy results in the increased frequency of phone calls and other means of contact. Veterans enrolled in the control group are contacted by the study team every two weeks over the eight-week period, corresponding with their counterparts randomized to the intervention arm. During the point of contact, the veteran in the supported discharge group answers the M2PI [65]. The M2PI is administered either through a secure online portal or over the phone. These interactions are audiorecorded.

##### Modifications, adherence & concomitant care

Participants in either group are free to discontinue participation any time. The principal investigator may need to end an individual’s participation before completion if it is deemed appropriate or in their best interest. To improve adherence to the intervention, participants who fail to attend intervention appointments are called three times to follow up on continuation in the study. After the third attempted phone call, a letter is mailed to the participant’s address asking him or her to contact the study team. If no response is received, the participant’s enrollment in the study is terminated. Letters are also sent between the second and third assessment sessions as a reminder of the upcoming assessment appointment and upon completion of the study.

Veterans who are currently evaluated at weekly meetings by the DC VAMC Polytrauma Interdisciplinary team for TBI-related skilled therapy management are not enrolled in the study until evaluation at team meetings has been completed.

#### Outcomes

COMPASS^goal^ outcomes, the result of hypothesis testing on collected data, hinge on the assessment of variables measuring veteran well-being and community integration pre-and post-intervention, with 3 month follow-up post-intervention. When assembling the study assessment battery, we considered the following rules: a) the relevance of the measure to the study’s specific aim and hypotheses; b) the balance of the proposed measures by modes of expression and assessment quality (self-reported assessment versus experimental technique versus observation by a clinician or a family member); c) the possibility of premorbid (e.g., pre-injury that resulted in mTBI) executive functioning evaluation and sequential charting of executive function over time as it returns to the standardized norm; and d) the possibility of linking performance at the baseline with executive performance over time, using reliable change models to determine whether the change meets the criteria for some a priori “recovery threshold.”

Core TBI measures (see Table 3) were suggested by the TBI Common Data Elements (CDE) work group and/or those that are used in community integration TBI studies and which support a multilevel approach to data analysis. The U.S. National Institute of Neurological Disorders and Stroke, the VA, the National Institute on Disability, Independent Living, and Rehabilitation Research, the Defense Centers of Excellence for Psychological Health and Traumatic Brain Injury, and the Defense and Veterans Brain Injury Center have co-sponsored the scientific initiative to develop CDEs for TBI research.

**Table 3:** COMPASS^goal^ assessment battery

Domain	Test	Description	Administered
Cognitive functioning and TBI severity	VHA TBI Comprehensive Evaluation screening tool [67]	This is a reliable measure for detecting cognitive impairment in service members. If the veteran does not have a CTBIE available in the medical record, questions 6–8 will be collected by an IRB-approved member of the Polytrauma team.	Exclusionary screening
Demographic information	Patient Information Form	8 questions about demographics	Exclusionary Screening
Mental health	M.I.N.I. International Neuropsychiatric Interview (M.I.N.I.) 7.0.0 [69–72]	This is a reliable measure of mental disorders from DSM-V.	Exclusionary Screening
Executive dysfunction	FrSBe [68, 78, 79]	46-items. Both self-report and family-report.	Exclusionary screening, T2, and T3
	Trail Making Test (TMT) [80, 81]	This is a measure of cognitive processing speed, mental sequencing, visual search and motor speed that consists of two components: Part A and Part B. Part A requires the subject to connect 15 encircled numbers that are randomly arranged on a page in numerical order. Part B requires the subject to connect 15 encircled numbers and letters in alternating order. The score provided is the time required to complete the task.	T1, T2, and T3
	Controlled Oral Word Association Test (COWAT) [82, 83]	This is a measure of word generation and is considered an executive functioning measure. Letter fluency requires the person to generate words that start with a specific letter. Each trial lasts one minute, and the score is based on the number of words provided.	T1, T2, and T3
PTSD-related functional impairment	Brief Inventory of Psychosocial Functioning (B-IPF) [88]	The B-IPF is a 14-item self-report instrument that measures functional impairment across several domains, including self-care, intimate relationships, familial relationships, work, friendship and socializing, parenting, and education. Each domain is composed of two questions. The first asks respondents to rate how much difficulty they have experienced in a domain during the past 30 days. The second question asks respondents to rate the amount of distress they have experienced related to those difficulties during the past 30 days.	T1, T2, and T3
TBI self-efficacy	TBI Self-efficacy Questionnaire [91]	A 15-item TBI-specific scale of self-efficacy with regard to trauma to measure individual abilities to manage TBI consequences.	T1, T2, and T3
Emotional status and resilience	Brief Symptom Inventory −18 (BSI-18) assessment [86, 87]	A paper-and-pencil scale that consists of 18 items to measure depression, anxiety, somatic concerns, and general distress. It is a 5-point rating scale and is part of the CDE set.	T1, T2, and T3
	Post-traumatic Stress Disorder Checklist for DSM-V (PCL-V) [84]	The presence of PTSD symptoms. PCL-V is a 20-item self-report scale updated for the DSM-V to measure stress signs, intrusive thoughts, avoidance, behavior, and arousal symptoms.	T1, T2, and T3
	CRIS (Fixed Form; Resnik L.) [93–95]	107 veteran-specific items assessing extent of participation, perceived limitation in participation, and participation satisfaction	T1, T2, and T3
	Social participation via ICF qualifiers: Return to work and/or to school as defined by the ICF framework [60]	Work status/educational attainment confirmed by a family member/immediate caregiver or another reliable source	T1, T2, and T3
Overall well-being	Libin Verbal-Pictorial Association Standardized Personality Test (LIS) [89, 90]	The purpose of the test is to explore the personal meaning of drawings made as a response to 15 standardized verbal concepts ^12^.	T1, T2, and T3
Global measure of well-being and life satisfaction	Satisfaction with Life Scale (SWLS) [96]	Self-report based on a 5-item scale	T1, T2, and T3

*Tests performed by attending clinician or as part of the routine care at the DC VAMC

In accordance with the study’s specific aims, we also considered psychosocial individual assessments that capture the subjective aspects of recovery, including executive functioning [68, 78–83], amount of stress (PTSD) [84], functional capacity [20, 85], emotional status (including depression and anxiety levels) [86–90], TBI self-efficacy [91], coping [92], community integration [93–95], and life satisfaction [96]. These measures have proven external validity and are commonly used in TBI studies (see Table 3). The behavioral assessment focuses on individuals’ performance of real-world tasks [85]. The community integration measures focus on different aspects of societal participation and individual productivity, including educational attainment and employment [93, 94]. These measures also have good construct validity and test-retest reliability, and importantly, they are sensitive to change. Most measures employ alternate forms or demonstrate a low practice effect, which makes them appropriate for repeated administration. We are also aware that the types of measures included are complementary, such that “recovery” on the objective measures might not reflect “recovery” on the subjective measures.

#### Participant timeline

Following consent procedures, individuals agreeing to participate in the study are asked a series of screening questions to ensure that they meet all inclusion/exclusion criteria. The first data collection takes place within the first 14 days after screening and enrollment into the project and is described in full in the Outcomes section. Veterans will be contacted by the study interventionist or supported discharge group leader within 7 days of completing the first assessment session, following randomization. Follow-up assessment sessions will be collected approximately 8–10 weeks after randomization, upon completion of either group, and 5 months after randomization (see Fig. 3).

#### Sample size

The selection criteria focus on veterans with mTBI such as those who are routinely seen at the TBI clinic at the DC VAMC. The sample size calculations are based on a *t*-test for comparisons of mean change in the Community Reintegration of Service Members (CRIS) subscale, one of the primary outcome measures, from pre- to post-intervention between two groups.

##### Hypothesis

H0: μ1 = μ2, where μ1 = the mean change in the CRIS subscale from time 1 to time 2 in the self-management group and μ2 = the mean change in the CRIS subscale from time 1 to time 2 in the supported discharge group

$$ \mathrm{H}1:\ \upmu 1\ne\ \upmu 2 $$

##### Power calculation

Power Analysis and Sample Size Software (PASS) [97] was used for the power calculation. The primary outcome of this study is the frequency of social interactions measured via the Extent of Participation subscale of the CRIS [94, 95]. The power calculation was performed based on the hypothesis listed above using a two-sided *t*-test with the assumption of normality. The level of significance was set at 0.05, and the power was set at 90 %. The effect size and standard deviation used for the power calculation were estimated based on the VA study [4]. Forty-four subjects in each group, for a total of 88 subjects, provided 90 % power to test an effect size of 0.71 with an SD of 7 for the difference in the CRIS subscale scores between groups, with α = 0.05 (two sided). After adjusting for 20 % loss to follow-up, we will recruit 55 subjects in each group of this study for a total of 110 subjects.

#### Recruitment

Participants are recruited from the DC VAMC at least three months after sustaining a TBI. Consecutive patients ramping down from multi-disciplinary care who meet the inclusion/exclusion criteria are notified of the study and approached for informed consent. Participants are also recruited using recruitment flyers, brochures, and letters. COMPASS^goal^ project team members participate in VA TBI clinic weekly meetings to discuss potential participants, i.e., veterans ramping down from multidisciplinary care, to approach for informed consent.

### Assignment of interventions

#### Allocation

##### Sequence generation

Participants are randomly assigned to either control or experimental groups with a 1:1 ratio (105 + 105) using a computer-generated randomization list based on Wei’s Urn randomization algorithm. This algorithm is used to randomize subjects to two or more treatment groups of equal sizes. Wei’s Urn randomization algorithm dramatically changes the group assignment probabilities based on the degree of imbalance to achieve a longitudinal balance between groups [66]. Randomization list was obtained using PASS [97].

##### Concealment mechanism

Participants are randomized by the research coordinator. Allocation concealment is ensured as the randomization list is maintained in a secure, locked folder and accessed only upon completion of T1 assessments. The coordinator is unable to see the next participant’s assignment until the participant is ready for randomization.

##### Implementation

A randomization list was developed by the biostatistician, for use after baseline assessments are complete. The coordinator receives the randomization list from the biostatistician and keeps it in a secure location. Upon completion of assessments, the coordinator reveals information about group allocation to the study participant and relevant study staff providing services in either group. Staff members responsible for future data collection are not allowed to receive information about the group allocation. Thereby, randomization will be completed without influence from data collectors or statisticians.

#### Blinding (masking)

Staff members performing all outcome assessments are blinded to group assignment throughout the study, and participants are asked at each assessment not to reveal their group assignment to data collectors. In spite of these precautions, data collectors may become unmasked by a remark made by a participant during an assessment or an error made by the study staff. To document such occurrences, data collectors complete the Data Collector Estimation of Treatment Condition Form on which they (a) check off whether they have been unmasked and (b) indicate which group they think the participant was assigned to, if they believe they are unmasked.

##### Emergency unblinding

We do not anticipate any situations that would require emergency unblinding because the study data collector does not provide clinical care for participants.

### Data collection, management, and analysis

#### Data collection methods

The assessments are outlined further in Table 3. The data collector prepares all necessary questionnaires in packet form prior to each assessment session. All questions are read to the participants and significant others, and their answers are recorded by the data collector. No instruments are self-administered for this study. Participants are provided with a copy of questions and corresponding responses so that they can easy follow along with the interview.

##### Retention

To ensure study retention, the team uses a motivational technique that has proven useful in participant recruitment and retention. An Institutional Review Board (IRB) approved Certificate of Participation template issued to express appreciation to participants and family members for participating in the study in comprehensible, “layman” language. This approach will not only keep participants engaged with the study but will also potentially create a study atmosphere that will motivate them to bring other potential participants to the study to benefit from the same experience. To encourage ongoing patient engagement between assessment sessions T2 and T3, veterans receive IRB-approved Engagement Templates as reminders of their COMPASS^goal^ participation. This outreach technique reminds participants that they are enrolled in the COMPASS^goal^ study without providing information that may influence study variables.

#### Data management

Study information management procedures protect the privacy and confidentiality of individually identifiable participant information. Handwritten notes of study personnel are kept in locked file cabinets in researchers’ offices at the DC VAMC. Data from primary source documents are entered and managed in a secured database and are regularly reviewed for quality and completeness by the study coordinator. The data are analyzed through SPSS IBM, v.21.0 [98] on a VA Informatics and Computing Infrastructure (VINCI) server [99]. If data are downloaded from VINCI to share with non-VA collaborators, the data are de-identified. VINCI is used to upload and store data, but no data are requested from the national databases supplied through VINCI. Files are maintained in accordance with the Records Control Schedule.

Electronic data entry forms reflect primary source documents to facilitate accurate data entry. Trained study staff enter data immediately following collection. Data integrity will be maintained through validation rules and calculated fields. Coding sheets are available for each assessment entered into the database to confirm the correct coding of non-numeric data. Periodically, 10 % of the data is checked by the study PI or a designated official to ensure proper data entry. High error rates will require more investigation of data entry procedures. All data will be analyzed for errors prior to analysis.

#### Statistical methods

##### Outcomes

For this project, we collect three major types of data assessed at times 1–3 (baseline/pre-intervention, post-intervention/supported discharge, and at three months post-treatment follow-up): community integration parameters, including educational attainment and employment and individual characteristics such as PTSD; TBI self-efficacy, emotional status, and coping, including resilience, performance of real-world tasks, and life satisfaction; and neuropsychological parameters, including overall cognitive and executive functioning. The primary outcome is a change in the three major types of parameters from pre- to post-intervention. Depending upon the hypothesis being tested (see Background: Objective, Specific Aim, and Hypotheses), participants are stratified according to their level of executive functioning, their severity of conditions secondary to TBI (e.g., PTSD, emotional status), or their degree of social participation. Dependent variables include TBI-self-efficacy, community integration indices, educational or work attainment as defined by the ICF qualifiers, and life satisfaction. Both objective (e.g., executive performance) and subjective (e.g., perceived TBI self-efficacy) data are then obtained based on repeated measures over the enrollment period. Multiple regression models will be fitted to explore continuous primary and secondary outcomes differentiating groups at each time point and changes at the intra-individual and inter-individual levels over time. For categorical outcomes (e.g., low/high executive performance or work status, such as unemployed/employed or part-time/full-time), unconditional logistic and/or multinomial regression models will be implemented.

The basic testing of the hypotheses will involve an Analysis of Variance (ANOVA) test and a repeated-measures ANOVA test. Bonferroni adjustments will be made to account for multiple testing.

##### Hypothesis 1: outcome variables main effects for community re-integration factors

c an interaction between the intervention and the control groups can be modeled as a fixed effect. A linear mixed model (LMM) [100] will be used to evaluate whether the intervention had an effect at any time point or whether the intervention influenced change (growth, trajectory) in the outcome over the course of the study. The following will be considered for the analytical strategy:

##### ANOVA

Repeated-measures ANOVA and multivariate analysis of covariance (MANCOVA) will be used to compare the effect of the COMPASS^goal^ intervention versus the supported discharge group, as determined by baseline assessment. ANOVA will be used to compare the change pre- to post-intervention between the two groups. Furthermore, to analyze the main effect of the COMPASS^goal^ intervention over time, a two-way repeated measures parametric ANOVA and a non-parametric repeated measures Friedman ANOVA will be performed using the baseline time point, levels of TBI self-efficacy, or real-world task performance as the dependent variables. The levels of the repeated-measures factor correspond to the number of assessments: baseline/pre-intervention, post-intervention/supported discharge, and at three months post-treatment follow-up. The between-groups factor will be the intervention group versus the supported discharge group.

The MANCOVA analysis will focus on the contrast in TBI self-efficacy between the intervention and supported discharge groups while accounting for a specified covariate: level of executive impairment, as defined by FrSBe. In the case of non-normal residuals, we will use a nonparametric repeated-measures approach. In the case of missing data, an LMM, which uses a maximum likelihood estimate to correct for an unequal number of measures per subject, will be employed [100].

##### Hypothesis 2: psychosocial profile as a mediator of the responsiveness to the intervention over the time course

To explore Hypothesis 2, we will utilize a multistage analytic strategy. Because of the possibility of missing data due to non-responses, missed visits, attrition, and mortality over the course of the study, the statistical analysis presents certain challenges. At the first stage, an LMM will be used to incorporate all available data, to evaluate trends, and to estimate changes in outcome variables without discarding cases that have missing data points. Furthermore, an LMM controls for confounding effects of other repeatedly measured covariates while accounting for the correlations among repeatedly measured outcomes [100]. SAS PROC MIXED [101] will be used to estimate an LMM for each outcome of interest. For categorical outcomes, generalized estimating equations (GEEs) will be used to evaluate trends over time while accounting for the dependency among the repeatedly measured outcomes. GEEs will be solved using SAS PROC GENMOD [101].

For Hypothesis 2 analysis, the following will also be considered:

- ○ An LMM will be used to control for the confounding effects of other repeatedly measured covariates while accounting for the dependency among the repeatedly measured outcomes and covariance matrix.
- ○ We will construct a model that includes only the repeated-measures variables to obtain the means, variances, and covariances.
- ○ We will add time-invariant variables such as the treatment group into the multi-level model (MEME) to predict the change over time in executive dysfunction and associated real-life task performance. This method will allow us to address individual growth, to identify latent trajectories of growth, to relate the observed changes to pre-existing differences between study participants, and to determine treatment effects.

Subsequently, we will use linear growth curves to assess individual differences and group differences following a two-stage linear growth model. At the second stage, we will construct a model that includes only the repeated-measures variables to obtain means, variances, and covariances. At stage three, we will add time-invariant variables such as age, gender, and education into the model to predict change over time in executive function and real- world performance. We will also be able to refine our model at any level by assessing the fit after additional variables are inserted. The covariance structure obtained will allow us to draw inferences with regards to linear increases or decreases in executive dysfunction and real-world performance over time. This method will allow us to address individual growth, to identify latent trajectories of growth, and finally, to relate the observed changes to pre-existing differences between study participants.

##### Additional analyses

We will use an LMM to control for potential confounding variables, and baseline values will be utilized as a covariate. These models will allow for the additional control of potential variance within subject clusters from variables, such as intervention modality. These clusters, which are not part of the formal hypothesis testing but are byproducts of these models, will allow for the conceptualization of additional future hypotheses.

##### Analysis population and missing data

Rigorous methods to address loss to follow-up and missing data are important. As in many studies with vulnerable populations, participant dropout or censoring may be informative. For example, sicker patients and those with sub-optimal treatment results may opt to discontinue participating or providing samples or questionnaire responses. Thus, the probability of missing outcome data may be dependent on covariate data and, hence, may be “non-ignorable.” [102] To assess the probable types of missing data, baseline covariates among patients with and without missing data will be compared. If missing data are judged as *missing completely at random*, the typical strategy will be to conduct a complete case analysis, recognizing a loss of precision. The exception to this strategy will be when considerable data (i.e., >15 %) are missing on a particular covariate that is judged to be critical for inclusion in the analysis. In this instance, imputation by unconditional or conditional mean imputation will be used; these simple approaches perform well when the overall percentage of missing data is low. In rare instances when the percentage of missing data is not low (i.e., >15 %), more sophisticated multiple imputation methods may be employed. Imputation methods will not be used to fill in values for missing outcome data.

### Monitoring

#### Data monitoring

##### Formal committee

A data monitoring committee has not been established because the study has been rated a minimal risk study by the local IRB.

##### Interim analysis

No interim analysis of the primary endpoint will be performed because this is a minimal risk study. All members of the study team have access to de-identified data while the trial is ongoing; however, masking is preserved for the data collector and statistician. The principal investigator and sponsor have the ultimate authority to stop or modify the trial.

#### Harms

For purposes of this study, adverse events are defined as any “untoward medical occurrence” temporally associated with the intervention, regardless of causality [103]. Adverse events are routinely monitored and tracked by the study staff. Any adverse event that meets the criteria for a serious adverse event, including death, hospitalization, or any event that jeopardizes the safety of the subject, will be reported to the IRB within 5 business days of the study team’s notification of the event. Monitoring for adverse events begins when the informed consent is signed and continues until 30 days after the participant completes the study or withdraws.

#### Auditing

The research compliance officer at the DC VAMC runs quarterly audits on informed consent and Health Insurance Portability and Accountability Act (HIPAA) documents. At each time point, a study coordinator supplies the compliance officer with a list of participants who consented and re-consented to the study during the previous quarter. The compliance officer schedules a time to examine the consent forms and HIPAA authorizations for completeness, and a report is issued. Every three years, the research compliance officer also completes a review of the study records to ensure ethics and scientific approvals are maintained appropriately.

## Ethics and dissemination

### Research ethics approval

The protocol, recruitment procedures, consent forms, and HIPAA authorizations were reviewed and approved by the DC VAMC IRB and Research & Development Committees prior to recruitment initiation. The PI provides annual progress reports to the IRB for continuing reviews annually.

### Protocol amendments

Any significant modifications to the study protocol, such as changes to the study population, design, or implementation procedures, will be submitted to the IRB for approval prior to implementation.

### Consent or assent

Once a potential subject is identified in the DC VAMC TBI database or through the TBI clinical team, a COMPASS^goal^ team member, researcher or clinician, as is appropriate to each veteran’s circumstances, approaches the veteran and/or family members about the study. Special care is taken to explain the nature of the study and all risks/benefits to the individual in language that is appropriate for his/her comprehension level. In addition, study investigators take special precautions to ensure that potential study participants fully understand the consent form and authorization for the release of protected health information (e.g., by reviewing the consent form and answering any questions the individual may have).

#### Ancillary studies

Data will not be stored in a data repository for use in ancillary studies.

### Confidentiality

Once written consent has been obtained, subjects are assigned a de-identified study number for data collection purposes by the PI. All collected data are stored in a secure, locked cabinet in a locked room at the DC VAMC and in a password-protected database. Consent forms/HIPAA authorizations are stored separately from the research record to maintain confidentiality. All crosswalk documents with personal health information to the de-identified research record are kept in a locked cabinet separate from the research record. Any discussion of identifiable patient data is sent via encrypted email. Protected health information, as defined by HIPAA, will never be used for any purpose other than the research activities of this study.

### Declaration of interests

The study team has no competing interests or conflicts of interest to report.

### Access to data

Only IRB-approved members of the study team will have access to the data collected in this study. If members of the research team leave the team, they will no longer have access to the data.

### Ancillary and post-trial care

The DC VAMC will provide necessary medical treatment if participants are injured as a result of their participation in this study, unless they were injured because they did not follow the instructions they were given. Eligibility for other VA services will not be affected by participation in this trial. Following completion of COMPASS^goal^, participants may continue to engage in medical care but will not continue to receive the COMPASS^goal^ intervention or support from the study team.

### Dissemination policy

#### Trial results

No publication restrictions are in place for this trial.

#### Authorship

Publications resultant from the COMPASS^goal^ study will comply with the International Committee of Medical Journal Editors [104].

#### Reproducible research

The study protocol will be disseminated to the general public through this publication.

## Results

At this writing, COMPASS^goal^ has begun testing and has enrolled 20 % of its target. The study COMPASS^goal^ Manual has been developed via the qualitative Phase I and is now implemented consistently during treatment sessions. See Fig. 3 to review the study execution algorithm.

## Discussion

### Limitations

The recruitment of participants from the mTBI population proved to be challenging initially; however, lessons learned from the early phases of recruitment strengthened and refined the study team’s approach. Originally, potential participants were required to have been discharged from interdisciplinary skilled therapies, which limited recruitment because many potential participants either re-engaged with therapy—and thereby were excluded—or ceased coming to the VA for appointments altogether upon discharge, making it difficult to recruit participants face-to-face, in accordance with VA IRB policies.

Following the identification of this problem, “discharge” from interdisciplinary treatment was more clearly defined as not being currently under review at weekly polytrauma team meetings for TBI-related skilled therapy management. Recruitment opportunities increased substantially and were subsequently further enhanced by IRB-approved flyers and letters.

### Generalizability

This project may be regarded as a first-stage project in which the efficacy of the method is demonstrated, and the efficiency of each component is tested. The next phase, which will follow this study, would involve the development of several projects aimed at exploring the mechanisms underlying the successful vs. non-successful adaptation of veterans to everyday life and routes for transferring the ongoing delivery of intervention to the daily routine of community-dwelling veterans with TBI.

The study will prepare the field of creating supportive care environments for necessary future studies on the utilization of best clinical practice guidelines for the everyday life of veterans and their families. Clinical and applied research will concentrate on the utility of goal self-management interventions in routine care in both home and institutional settings, such as VA hospitals and community centers. These studies will also examine the cost benefits of this approach, which are expected to be substantial because the approach will lessen the time health service providers (e.g., TBI case managers or TBI social workers) must devote to their clients. In the basic research venue, studies need to explore the links between neuroanatomical structure, executive function, and everyday problem-solving in the context of psychosocial interventional research. This research would not only help improve the quality of life for veterans with TBI and other neurological deficits but also have important social benefits, such as training relatives and immediate caregivers on how to engage their loved ones in meaningful, healthy, and productive activities. The methodology proposed could be utilized with technology-based and other non-pharmacological interventions to better understand the needs and preferences of different clinical populations with chronic illnesses, or physical, cognitive, and behavioral disabilities (i.e., persons with PTSD, substance abuse, disturbing behaviors, and suicidal ideation). This particular extension may elucidate the commonalities and differences among various clinical groups and provide additional tools to improve the individual care of returning veterans.

## Abbreviations

ANOVA: analysis of variance
B-IPF: Brief Inventory of Psychosocial Functioning
BSI-18: Brief Symptom Inventory-18
CDE: common data elements
CNS: central nervous system
COMPASS^goal^: Community Participation through Self-Efficacy Skills Development
COWAT: Controlled Oral World Association Test
CRIS: Community Reintegration of Service Members
DC VAMC: Washington DC VA Medical Center
EFPT: Executive Function Performance Test
EFS: executive functional system
FrSBe: Frontal Systems Behavior Scale
GEE: generalized estimating equation
ICF: International Classification of Functioning, Disability, and Health
HIPAA: Health Insurance Portability and Accountability Act
IRB: Institutional Review Board
LIS: Libin Verbal-Pictorial Association Standardized Personality Test
LMM: linear mixed model
M2PI: Mayo-Portland Adaptability Inventory – 4, Participation Index
MANCOVA: multivariate analysis of covariance
MEME: multi-level model
M.I.N.I.: M.I.N.I. International Neuropsychiatric Interview
mTBI: mild traumatic brain injury
PASS: Power Analysis and Sample Size Software
PCL-V: Posttraumatic Stress Disorder Checklist for DSM-V
PTSD: post-traumatic stress disorder
RAND: research and development
RCT: randomized controlled trial
SWLS: Satisfaction with Life Scale
TBI: traumatic brain injury
TMT: Trail Making Test
U.S.: United States
VA: Department of Veterans Affairs
VHA: Veterans Health Administration
VINCI: VA Informatics and Computing Infrastructure
